# A Novel 3D Culture Model of Human ASCs Reduces Cell Death in Spheroid Cores and Maintains Inner Cell Proliferation Compared With a Nonadherent 3D Culture

**DOI:** 10.3389/fcell.2021.737275

**Published:** 2021-11-11

**Authors:** Liang Luo, Wei Zhang, Jing Wang, Ming Zhao, Kuo Shen, Yanhui Jia, Yan Li, Jian Zhang, Weixia Cai, Dan Xiao, Xiaozhi Bai, Kaituo Liu, Kejia Wang, Yue Zhang, Huayu Zhu, Qin Zhou, Dahai Hu

**Affiliations:** ^1^ Department of Burns and Cutaneous Surgery, Xijing Hospital, the Fourth Military Medical University, Xi’ an, China; ^2^ Department of Plastic and Aesthetic Surgery, The First Affiliated Hospital of Xi’an Medical University, Xi’an, China

**Keywords:** 3D culture, organoid, ASCs, 3D-ASC, transdifferentiation

## Abstract

3D cell culture technologies have recently shown very valuable promise for applications in regenerative medicine, but the most common 3D culture methods for mesenchymal stem cells still have limitations for clinical application, mainly due to the slowdown of inner cell proliferation and increase in cell death rate. We previously developed a new 3D culture of adipose-derived mesenchymal stem cells (ASCs) based on its self-feeder layer, which solves the two issues of ASC 3D cell culture on ultra-low attachment (ULA) surface. In this study, we compared the 3D spheroids formed on the self-feeder layer (SLF-3D ASCs) with the spheroids formed by using ULA plates (ULA-3D ASCs). We discovered that the cells of SLF-3D spheroids still have a greater proliferation ability than ULA-3D ASCs, and the volume of these spheroids increases rather than shrinks, with more viable cells in 3D spheroids compared with the ULA-3D ASCs. Furthermore, it was discovered that the SLF-3D ASCs are likely to exhibit the abovementioned unique properties due to change in the expression level of ECM-related genes, like COL3A1, MMP3, HAS1, and FN1. These results indicate that the SLF-3D spheroid is a promising way forward for clinical application.

## Introduction

For 3D stem cell culture, the 3D cell environment can be manipulated to mimic what a cell experiences *in vivo* and provide more accurate information on cell interactions, metabolic profiling, and other cell normal physiology data; moreover, this culture has more valuable applications in stem cell research, drug discovery, tumor therapy, and regenerative medicine (Duval et al., 2017). Through further development and improvement, 3D cell culture is also likely to become an alternative method for studying organ behavior; for example, the current very promising organoid culture is expected to eventually bridge the gap between 2D cell culture and *in vivo* models (Belfiore et al., 2021).

At present, 3D cell culture technologies mainly have two types of methods: scaffold and scaffold-free techniques (De Pieri et al., 2021). Scaffold-based techniques such as hydrogel scaffolds, paper-based culture, fiber scaffolds, and other synthetic biological materials are employed, each of which has its advantages and applications (Pennarossa et al., 2021). Another method is scaffold-free techniques, such as the hanging drop method, magnetic levitation, and the ultra-low attachment (ULA) surface of culture vessels (Shen and Horbett, 2001; Ovsianikov et al., 2018). Among them, the ULA-3D culture technique, because of its inexpensive, simple, and easily operated properties, is currently widely used in general laboratories.

Adipose-derived stem cells (ASCs) have shown great potential in regenerative medicine, as they have a wide range of sources and have multilineage differentiation potential, low immunogenicity, and self-renewal ability (Zuk et al., 2002; Baptista, 2020). Typically, ASCs proliferate and expand on the surface of a tissue culture-treated dish such as a 2D monolayer. However, ASC expansion on the flat layer of the plastic surface cannot accurately mimic the natural microenvironment for cell growth *in vivo* due to a lack of three-dimensional tissue structure information (Yin et al., 2020). Furthermore, more evidence has shown that the extensive passage of ASCs in planar culture can cause cell morphology changes, leading to cell cycle arrest, induction of replication and senescence, and loss of differentiation potential (Delben et al., 2021; Jeske et al., 2021). As a result, scientists are trying to find new cultivation methods that can overcome or partially overcome these problems, and 3D ASC culture is a novel approach.

Several studies have been devised to culture 3D cell spheroids of ASCs (Di Stefano et al., 2020; Hoefner et al., 2020; Sung et al., 2020; Yin et al., 2020). In these studies, ULA-3D culture has been more widely used, and in addition to its simplicity and inexpensive nature, this method has the following advantages: it is compatible with many cell lines, initiates by self-assembly, and consists of natural cells and their deposited extracellular matrix (ECM) (Vu et al., 2021). These methods are also more suitable for biological analysis methods that solve basic scientific issues, such as the regulation of protein abundance due to changes in the 3D environment. However, scaffold-free culture methods have often been associated with decreases in cellular proliferation and viability (Fitzgerald et al., 2020; Hoefner et al., 2020); furthermore, uncontrolled cell assembly in the method often results in large differences in the size of the spheroids. These issues have limited the 3D ASC spheroid methods applied in the clinic.

In previous experiments, we discovered that ASCs cultured with low concentrations of FBS and growth factors (such as bFGF, EGF, and PDGF) can form the 3D spheres on specific matrix scaffolds; the special matrix is formed by a subset of plastic-adherent ASCs. The 3D spheroids based on a self-feeder layer (SLF-3D) have been proven to have multilineage differentiation potential and “stemness” properties (Luo et al., 2021; Supplementary Figure S1); they can survive for at least 10 passages and still could proliferate. This situation is very different from that of 3D cell spheroids generated by the ULA method. Therefore, in the following experiments, we compared the novel 3D culture (SLF-3D) method with the ULA-3D method to determine whether this method can overcome some of the main issues of the traditional ULA method.

In this study, we characterized the two methods for 3D ASC culture and compared the properties of SLF-3D spheroids with the ULA-3D spheroid. The related properties of SLF-3D spheroid ASCs, including spheroid morphological analysis, spheroid viability, and cell live/dead assay, were investigated by stereology, fluorescence technique, and flow cytometry. We further studied the possible mechanisms for the difference between SLF-3D and ULA-3D based on the expression level of ECM genes. We hope that the self-feeder layer 3D culture can be used as a simple and effective ASC culture optimization strategy to meet clinical needs.

## Materials and methods

### Monolayer Culture of Human ASCs

All human adipose tissue-derived stromal cells (ASCs) used in the present study come from freeze isolated cells; the process of ASC isolation was performed as previously described (Luo et al., 2021, 3). In this study, no animals were used, and all experiments were performed at the cellular level.

In the first two or three passage cultures using the traditional monolayer culture method, the cells were plated at 200 cells/cm^2^ densities (Lund et al., 2009) in T75 flasks (Corning) and cultured in ASC culture medium DMEM/F12 supplemented with 10% fetal bovine serum (FBS, AusGeneX) at 37°C with 5% CO_2_ at saturating humidity. When cells reached about 80–90% confluency, the cells were detached with Accutase (Life Technologies) about 3–5 m and the Accutase was inactivated with serum-containing media.

### Three-Dimensional Adipose Stem Cell Spheroid Culture

For 3D self-feeder ASC spheroid (SLF-3D ASC) cultivation: after 2D monolayer culture, the single-cell suspensions were produced by incubation Accutase reagent (Life Technologies) dissociation for 5 min at 37°C with gentle shaking and rinsed with 1× PBS. These ASCs were plated in a 3D culture medium at high density (5 × 10^4^ cells/cm^2^) and were cultured for more than 5 days, and the main components of 3D specific-condition culture medium are DMEM/F12 supplemented with 3% FBS (AusGeneX), human basic fibroblast growth factor (bFGF, 5 ng/ml, Peprotech), human epidermal growth factor (EGF, 2 ng/ml, Peprotech), human PDGF (5 ng/ml, Peprotech), heparin (2 μg/ml, Sigma), L-Ascorbic acid-2-phosphate sesquimagnesium salt hydrate (50 μg/ml, Sigma), 100 units/ml penicillin, and 100 pg/ml streptomycin. FBS should be tested by batch-to-batch assay before use and must ensure that FBS is enough to support the survival of cells.

**FIGURE 1:**
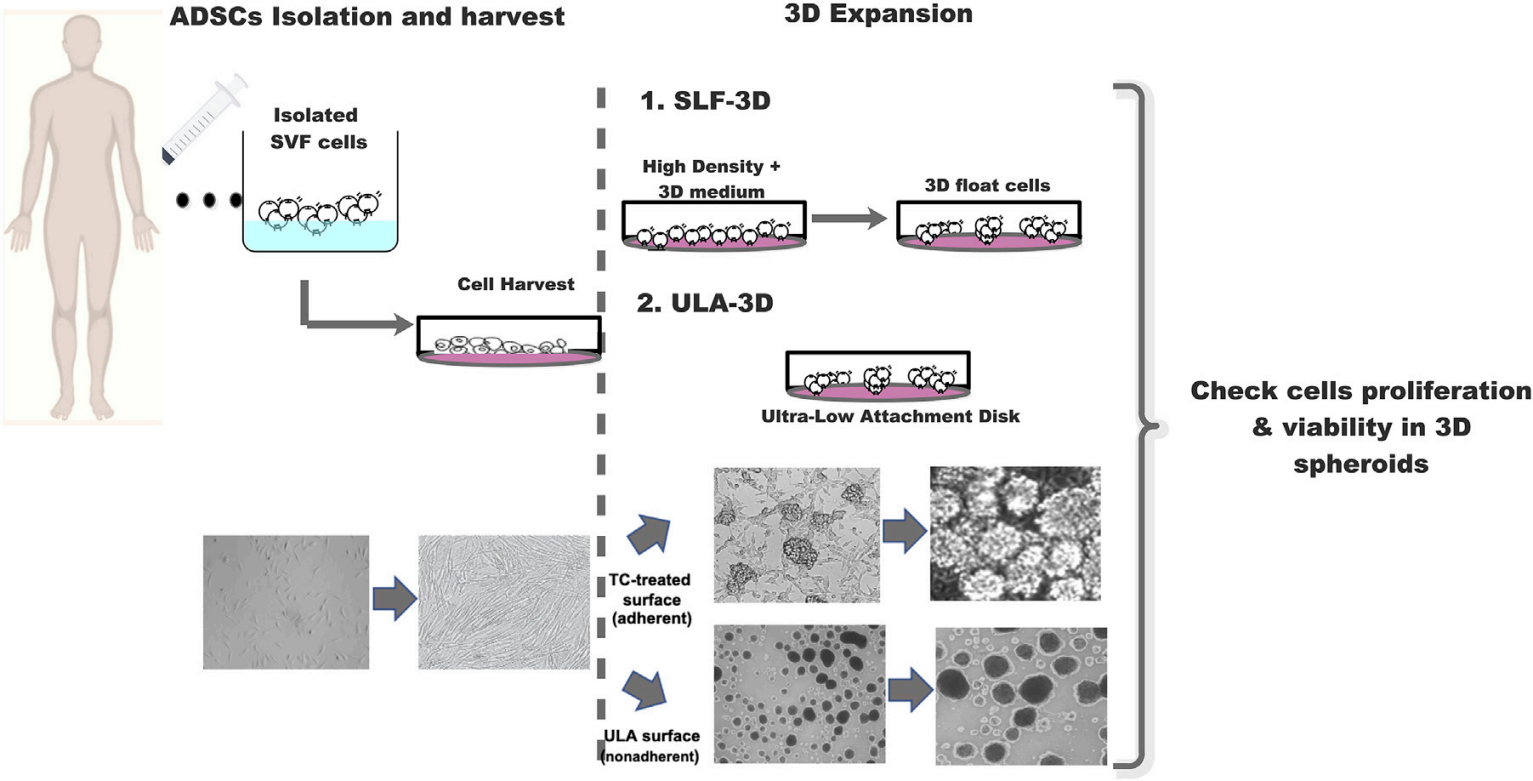
Schematic diagram of the strategy for 3D-cultured adipose stem cells. Schematic diagram SLF-3D form spheroids and assay method. Morphological observation of 3D spheroid formation. Phase-contrast images of cell expansion at low density, high density, and 3D spheres.

After the ASCs grow to 90–100%, they will form semi-suspended spheroids on the attached ASCs, and we called these cells 3D-ASC spheroids (SLF-3D). The medium was replenished with fresh medium every 2 days. The culture procedure is depicted in Figure 1. When more than 10 spheroids are formed per square centimeter, primary spheroids were collected by vigorous shaking and centrifugation at 100 × *g* for 3 min and cell spheroids were dissociated into single cells with Accutase for 5–8 min followed by another passage or and ready for follow-up experiments. For another passage, these single cells were reseeded into the original flasks, where some attached ASCs remained cells were cultured for approximately 1 week, and this process was then repeated for each passage.

For ultra-low attachment 3D spheroid (ULA-3D) cultivation: the cells were also passaged by utilizing a general cell passage protocol. The ASCs thus prepared were resuspended in ULA dishes (Corning Incorporated, United States, #3471) in DMEM supplemented with 10% FBS at 37°C in a 5.0% CO_2_ incubator and were utilized for later assays.

### Spheroid Viability

For ULA-3D ASC spheroid viability assay, 10,000 or 5,000 ASCs were seeded in ULA 96-well plates (Corning Incorporated, United States, #3474) that were centrifuged 5 min at 4°C at 200 × *g*. Furthermore, for SLF-3D spheroid viability assay, we first collected SLF-3D spheroids (at 3 days) with the same sedimentation speed in tubes by natural gravity, and as these spheroids contained subpopulations that differed in cell volume, and these subpopulations could be isolated according to their sedimentation velocity at unit gravity, dilute these collected spheroids to a 96-well plate for secondary selection and then select the appropriate size spheres in the 96-well plate for subsequent related experiments. All spheroids were cultivated in a humidified atmosphere containing 5% CO_2_ at 37°C for a period, as indicated in each experiment.

After 3, 5, and 7 days of cell culture, spheroids were incubated for 30 min at 37°C with 2 μM Calcein-AM and PI (LIVE/DEAD viability/cytotoxicity kit, Invitrogen). In living cells, active intracellular esterase cleaves the Calcein-AM to intensely fluorescent Calcein, which is retained within cells with membrane integrity. Fluorescence was observed using a Zeiss microscope (Zeiss Observe.A1 Axio).

### Cell Counting Kit-8 Assays

Cell proliferation was measured using the Cell Counting Kit-8 (CCK-8, Sangon, Shanghai, China). SLF-3D and ULA-3D spheroids (which were dissociated into single cells with Accutase) were plated into a 96-well microplate with 10% FBS DMEM/F12 medium at a density of 1,000 cells per well. At 24, 48, 72, and 96 h, CCK-8 reagent (10 µl per well) was added to the cells and then incubated at 37°C for 2 h, and then the growth curves of cells were generated using absorbance values detected using a microplate spectrophotometer (Tecan, Switzerland) at 450 nm.

To count the cells, the living and dead cells of SLF-3D and ULA-3D spheroids (which were dissociated into single cells with Accutase) were counted using either the Countess^®^ Automated Cell Counter (Invitrogen) or hemocytometer and using Trypan blue (Invitrogen) exclusion.

### Measurement of Viability Spheroid Cells by Flow Cytometry

The SLF-3D and ULA-3D cells (which were dissociated into single cells) were collected by centrifugalization and transferred to EP tubes. Flow cytometry was performed using the Annexin V-FITC Apoptosis Detection Kit (BD Biosciences) following the manufacturer’s instructions, and cells were analyzed using a BD Accuri™ C6. In flow cytometry analysis, >10,000 events were measured per sample, and unstained cells were used as controls. All data were analyzed by Accuri™ C6 and Prism 8 (mac OS version) software.

### RNA Extraction and Quantitative Real-Time RT-PCR Analysis

RNA was isolated from cells using TRIzol (Invitrogen) according to the manufacturer’s protocol. Total cellular RNA was extracted from cell spheroids using TRIzol followed by treatment with RNase-free DNase according to the manufacturer’s protocol. To determine the expression levels of mRNA, total RNA was reverse transcribed with a PrimeScript^®^ RT Reagent Kit (TaKaRa). Approximately 500 ng of total RNA was used for the first-strand cDNA synthesis. Quantitative real-time RT-PCR was carried out using CFX Connect System (BIO-RAD) and subsequently amplified using the SYBR Green PCR Master Mix (TaKaRa) and 0.5 µM each of the sense and antisense primers. After amplification, the melting curves of the RT-PCR products were acquired to demonstrate product specificity. Results are expressed relative to the housekeeping gene GAPDH. Primer sequences are summarized in Supplementary Table S1.

### tatistical Analysis

The SPSS statistical software package (Chicago, IL) was used for statistical analysis. All experiments were performed at least in triplicate. Data were presented as mean ± standard deviation (SD). Comparisons were accomplished by one-way analysis of variance (ANOVA) with LSD post-hoc test or Student’s *t*-test. A statistical significance was defined as *p* < 0.05.

## Results

Previously, we developed a novel and economical self-feeding 3D spheroid (SLF-3D) culture method for adipose stem cells (more details are given in *Materials and Methods*, Figure 1) in which the semi-suspended spheroids are formed on adherent ASCs attached to plastic plates, where the adherent cells play roles in support of the semi-suspended 3D-ASC spheroids. To determine the characteristics of the SLF-3D method, we used the classic ultra-low attachment (ULA-3D) method as control and conducted the following experiments:

### Characterization of SLF-3D Cell Spheroids

Morphologically, most SLF-3D spheroids were semi-suspended, and these spheroids were the more regular size and more consistent in shape. In comparison, ULA-3D spheres were suspended with few adherent cell spheres, and they had more irregular sizes and a few large clumps.

**FIGURE 2:**
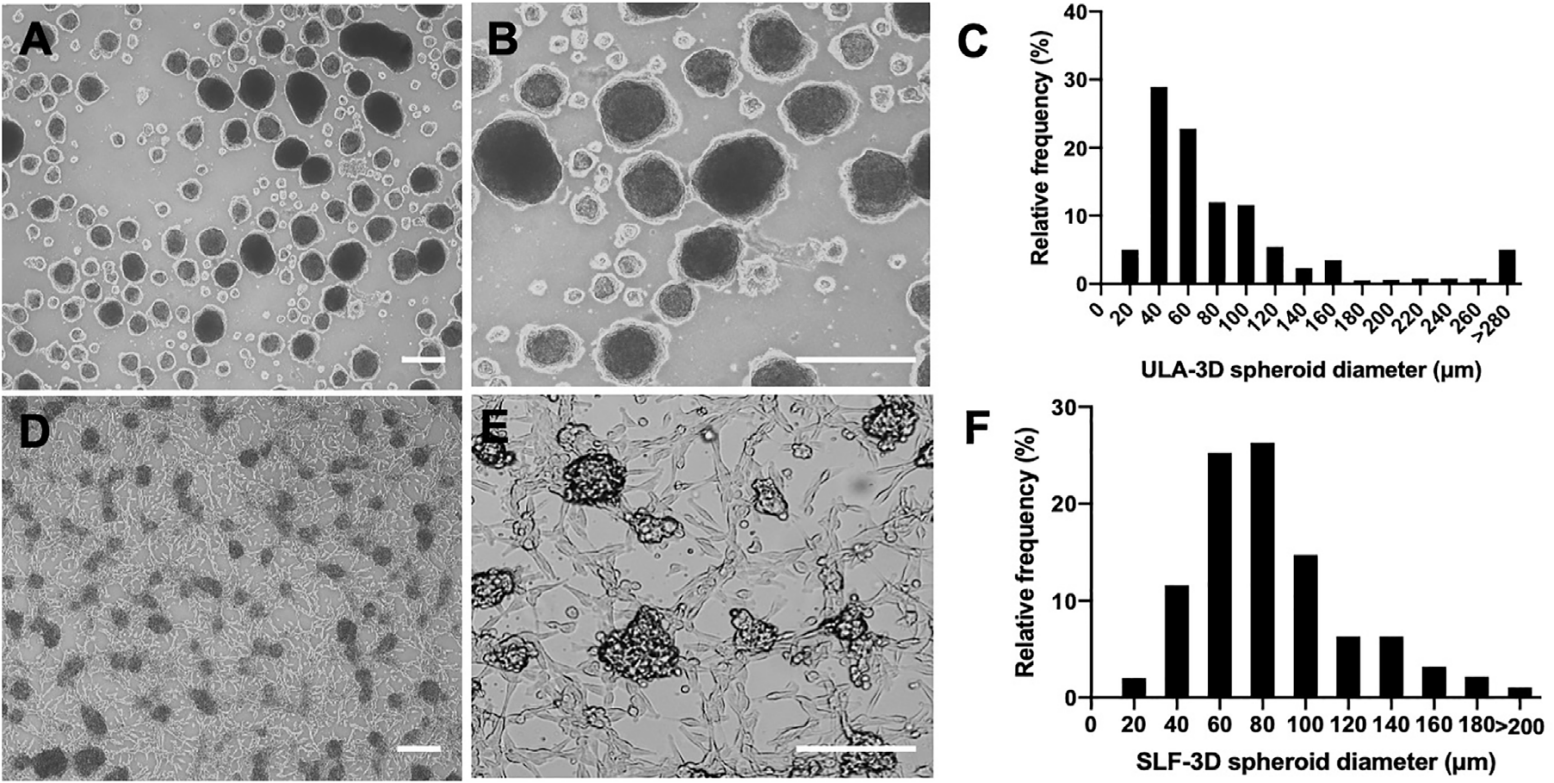
Comparison of the diameter distribution of the two methods (SLF-3D and ULA-3D spheroid methods). The morphology and distribution of spheroids generated with the self-feeder 3D (SLF-3D) culture method compared with spheroids from the traditional ultra-low attachment 3D (ULA-3D) method were compared. **(A,D)** Morphology of ULA-3D spheroids **(A,B)** after 3 days and SLF-3D spheroids **(D,E)** after the method described above (scale bars, 100 μm). **(C,F)** Spheroid diameter frequency distribution diagram. ULA-3D spheroids **(C)** and SLF-3D spheroids **(F)**. The spheres displayed heterogeneous sizes (scale bars, 100 μm).

The ULA-3D spheroids were found to be round with defined borders and varied in size 3 days after their first emergence (Figures 2A,B). As shown in Figure 2, the majority of the ULA-3D spheroids had a diameter of 20–40 μm, and the majority of the SLF-3D spheroids had a diameter of 60–80 μm (Figures 2D,E). ULA-3D spheroids with a diameter <20 μm or >280 μm represented approximately 10% of the spheroids formed; correspondingly, SLF-3D spheroids with a diameter of <20 μm or >200 μm represented less than 2% of the spheroids. Statistically, 85.3% of the SLF-3D cell spheroids mainly ranged between 20 and 100 μm in diameter, while 80% of the ULA-3D spheroids ranged between 20 and 160 μm in diameter, as observed from the frequency distribution diagram (Figures 2C,F).

It can be concluded from Figure 2 that spheroids prepared from ULA plates showed a wide size distribution. Spheroids with a diameter ranging from 100 to 200 μm are typically preferred in spheroid formation systems due to the low possibility of hypoxia and necrosis since the shrinkage of the cell spheroids causes a decrease in the oxygen supply to the sphere core, which is one of the main factors for the increase in cell necrosis of spheroids (Hirschhaeuser et al., 2010).

**FIGURE 3:**
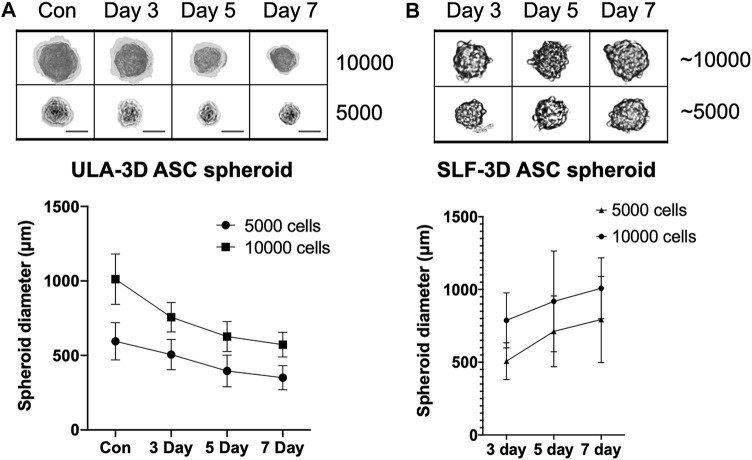
The relationship between culture time and spheroid diameter changes in the two types of spheroids. The relationship between seeding densities and the formation of **(A)** ULA-3D spheroids and **(B)** SLF-3D spheroids. Human ASCs were seeded onto ULA plates at densities of 10,000 and 5,000 cells/cm^2^. Scale bars, 100 μm. Images are representative of more than five independent experiments. Graphs show the means of three independent experiments, each performed in duplicate ±SE. *n* = 5; **p < 0.01.

To detect the volume change of SLF-3D spheroids with a limited number of cells, we used Corning ULA culture plates to form ULA-3D cell spheroids composed of 5,000 and 10,000 cells (Figure 3A). Meanwhile, the SLF-3D spheroids we collected were similar in diameter to the ULA-3D spheroids; specifically, the SLF-3D spheroids were collected after 3 days and a limited dilution to one to two spheres/100 μl was performed (Figure 3B), after which they were placed in an ultra-low adsorption culture plate and cultured in a proliferation medium for the comparison of these two kinds of spheroids by calculating the change in spheroid diameter.

The results showed that after 24 h, the diameter of the ULA-3D cell spheres formed by 10,000 cells was 1013 ± 169 μm (Figure 3A), while the diameter of the ULA-cell spheres formed by 5,000 cells was 595 ± 126 μm. After 3 days, there were basically no single cells in any well of the 96-well cell plate. Statistical analysis revealed that the ULA-3D cell spheres had average sphere diameters of 756 ± 99 μm, 627 ± 101 μm, and 572 ± 83 μm at 3, 5, and 7 days (Figure 3A), respectively, while the SLF-3D cell spheres corresponding to 10,000 cells had average initial diameters of 787 ± 189 μm, 918 ± 346 μm, and 1,008 ± 209 μm after 3, 5, and 7 days, respectively (Figure 3B). In addition, the self-3D cell spheres corresponding to 5,000 cells had an average spheroid diameter of 507 ± 127 μm at 3 days, and the diameters were 712 ± 244 μm and 793 ± 296 μm at 5 and 7 days, respectively (Figure 3, Supplementary Figure S3).

### Cell Viability of the SLF-3D and ULA-3D ASC Spheroids

The difference in volumes depends on the different growth rates of the spheroids. To confirm the difference in cell spheroid growth status between the SLF-3D and ULA-3D spheroids, we assessed spheroid proliferation within 96 h in 96-well plates.

**FIGURE 4:**
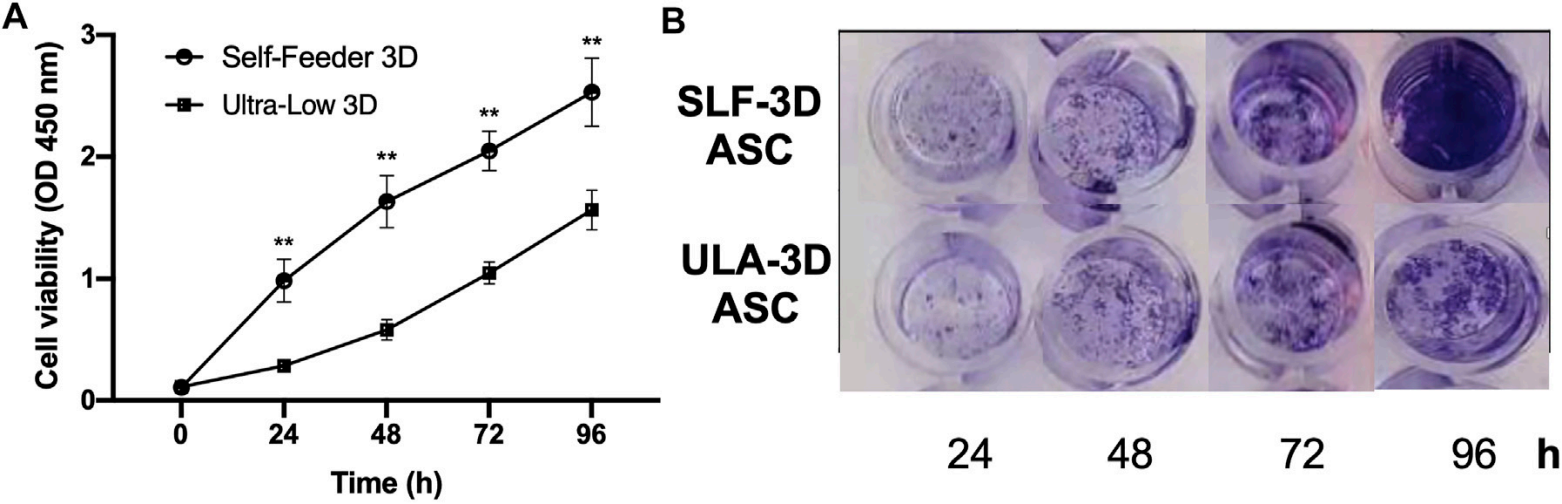
Cell viability of ASCs in SLF-3D and ULA-3D cultures at different time points. **(A)** Cell counting kit 8 (CKK-8) assay. Cell counting plot for ULA-3D and SLF-3D spheroids. Graphs show the means of three independent experiments, each performed in duplicate ±SE. *n* = 5; **p < 0.01, as compared with control (0 h) group. **(B)** Crystal violet stain of SLF-3D and ULA-3D ASC spheroids. **(A)** After 24, 48, 72, and 96 h, the spheroids were stained with crystal violet and photographed. Crystal violet staining: Cells were stained with crystal violet solution for 10 min. Three independent experiments were performed in duplicate, and representative results are shown. Scale bar, 100 μm.

Two types of spheroids were collected on the seventh day, and the Accutase was used to dissociate the spheroids into single cells, which were counted and then seeded in 96-well plates. Compared with the ULA-3D cells, the SLF-3D cells proliferated actively. The number of SLF-3D cells was significantly higher than the number of ULA-3D cells at 24, 48, 72, and 96 h (Figure 4). The result of crystal violet-stained cells was similar to the result of the measurement of viable cells (Figure 4B). The proliferation rate of the control cells was increased significantly from 24 h onward compared to that of cells under ULA culture conditions (Figure 4). Therefore, the low proliferation rate of ULA-3D cells is likely due to their limited growth capabilities.

### The Cell Viability of 3D Spheroids

**FIGURE 5:**
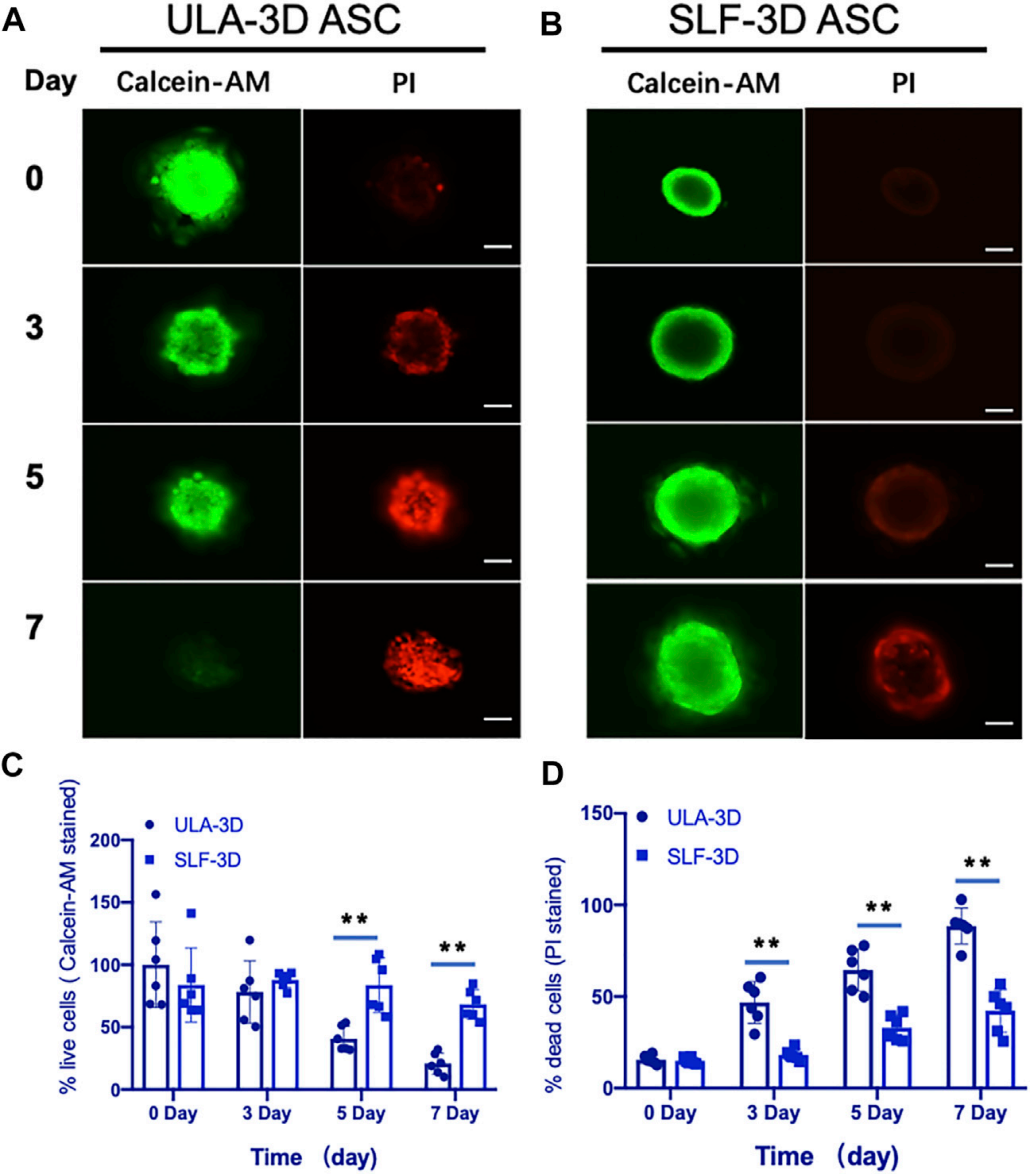
Fluorescence microscopy images of ULA-3D and SLF-3D spheroids stained with Calcein-AM/PI dyes. At 0, 3, 5, and 7 days of spheroid culture, spheroids were stained with Calcein-AM/PI. **(A)** ULA-3D spheroids; **(B)** SLF-3D spheroid ASCs, and the quantification of the percentage of **(C)** Calcein-AM positive and **(D)** PI-positive cells in images of spheroids. The mean integrated optical density (IOD) of all images was measured and analyzed using Image-Pro Plus software (*n* = 6), **p < 0.01, as compared with control (0 h) group. Scale bar, 100 μm.

To further determine the proportion of dead cells in the cell spheres, we performed staining with Calcein-AM and propidium iodide (PI) and visualized cell death within the sphere more directly through a fluorescence microscope. As shown in Figure 5, compared with the ULA-3D spheroids, only a few dead cells in the proliferating SLF-3D cell spheroids were stained by PI at 7 days (Figure 5B). The significant reduction in cells stained with Calcein-AM proved that the number of live cells in ULA-3D was reduced. At the same time, more cells in the cell sphere could be stained by PI, which also showed that there were more dead cells in the ULA-3D cell sphere (Figure 5A).

### Quantification of Spheroid Live/Death by Flow Cytometry

**FIGURE 6:**
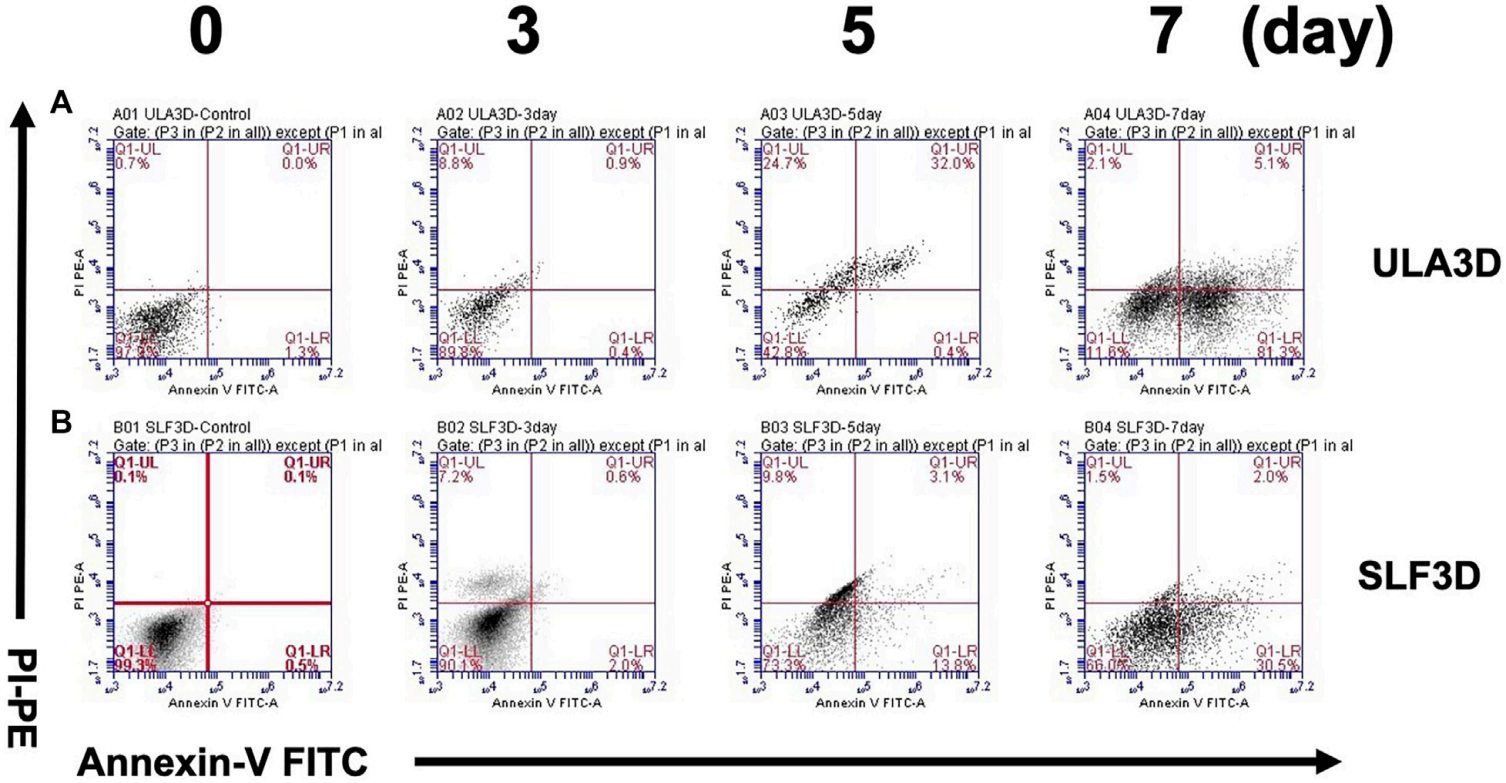
SLF-3D and ULA-3D spheroid cell viability was evaluated by flow cytometry. Viability of cells in the SLF-3D and ULA-3D spheroids. **(A,B)** Viability of ASCs as determined by flow cytometry measuring PI uptake and annexin V-FITC labeling. Representative log fluorescent dot plots and summary of the data are shown. As in the figure, 0, 3, 5ays, and 7 days indicate the number of days of cell culture. Cell populations were distinguished as live cells (PI-/annexinV-, lower left), or necrotic/dead/apoptotic cells (early apoptotic cells (PI−/Annexin V+), shown in the lower right; late apoptotic/dead cells (PI+/Annexin V+), shown in the upper right, and necrotic cells (PI+/Annexin V−), shown in the upper left. Three independent experiments were performed in duplicate, and representative results are shown. Data were acquired using BD Accuri C6 software.

To further validate the rate of cell viability in SLF-3D and ULA-3D spheroid cells, the flow cytometry method was used. Cell viability was assessed in SLF-3D and ULA-3D cultures at 0, 3, 5, and 7 days. The SLF-3D and ULA-3D cell death were comparable at 0 and 3 days under both culture conditions, and the highest number of dead cells occurred at 7 days (Figure 6). Particularly, the cell death (including necrotic/dead/apoptotic cell) rate in the ULA-3D culture (88.5%) was greater than that in SLF-3D culture (34.0%) at 7 days (Figure 6); in brief, the ULA-3D spheroids had a significant increase in the cell death population and a reduction in the live cell number compared to SLF-3D spheroids.

### Evaluation of ECM-Related Gene Expression in Spheroids

**FIGURE 7:**
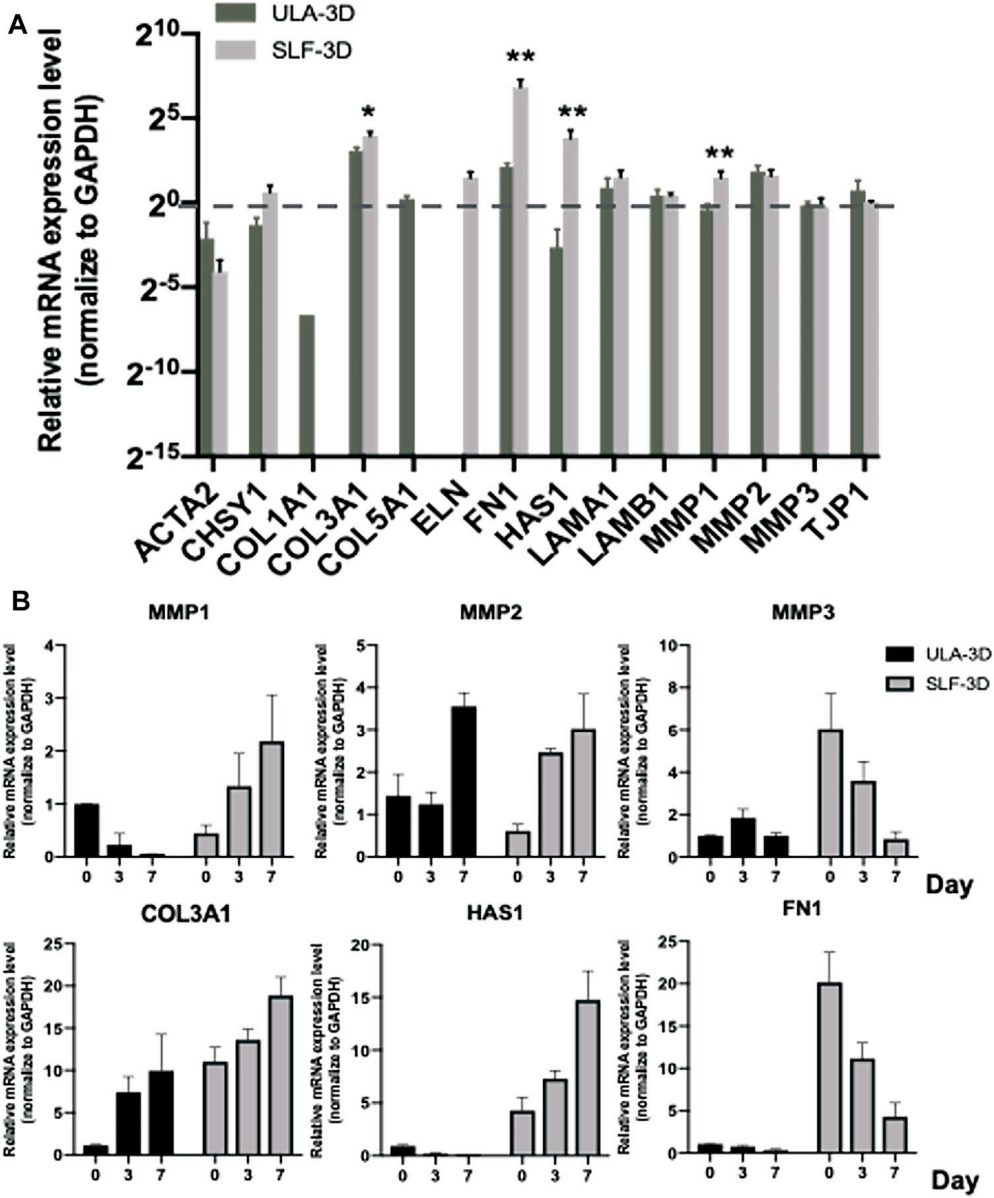
ECM-related gene expression level of SLF-3D and ULA-3D spheroid. **(A)** ECM-related gene expression changes were evaluated by quantifying the mRNA level at 7 days in SLF-3D and ULA-3D ASC spheroids. **(B)** Spheroids were generated from SLF-3D and ULA-3D ASC spheroids at 0 days (12 h), 3 days, or 7 days and used for qPCR assay. Graphs show the means of three independent experiments, each performed in duplicate ±SE. *n* = 3; **p < 0.01.

The ECM substitutes can profoundly alter cell growth, proliferation, and other cellular behaviors (Marastoni et al., 2008; Selman and Pardo, 2021). ECM-related gene expression changes (ACTA2, CSHY1, COL1A1, COL3A1, COL5A1, ELN, FN1, HAS1, LAMA1, LAMB1, MMP1, MMP2, MMP3, and TJP1) were evaluated by quantifying the mRNA level; we first screen out four genes with significant differential gene expression from common ECM-related genes at 7 days in SLF-3D and ULA-3D ASC spheroids, as shown in Figure 7A: COL3A1 (*p* = 0.01796), FN1 (*p* = 0.008601), HAS1 (*p* = 0.011761), and MMP1 (*p* = 0.011820); the expression level of these genes in the SLF-3D group was significantly higher than in the ULA-3D group.

Furthermore, we checked the expression level of these genes and their homologous genes in SLF-3D and ULA-3D spheroids on 0, 3, and 7 days, respectively (Figure 7B). The trend of the expression level of MMP1, MMP3, HAS1, and FN1 was significantly different between the two groups during 7 days. Among them, the expression level of MMP1 and HAS1 in SLF-3D spheroids increased and the expression trend was different from the ULA-3D group (Figure 7B). Furthermore, the expression levels of MMP3 and FN1 in SLF-3D spheroids drastically declined; at the same time, there was no significant difference in the expression levels of these two genes in the ULA-3D spheroids during 7 days (Figure 7B).

In short, these results revealed that SLF-3D ASCs are likely to exhibit the abovementioned unique properties due to the different expression patterns of these key ECM genes.

## Discussion

3D cell culture and the resulting organoid culture technology have recently shown promise for applications in regenerative medicine. ASCs have become a prospective stem cell source for clinical cell-based therapy (Fesharaki et al., 2018; Wang et al., 2019). At present, the 3D culture of ASCs, especially ULA-3D culture, is still difficult to apply for a large-scale application, and one of the important reasons for this is that the necrotic cells in the core of 3D spheroids increase rapidly due to the prolonged culture time.

Under normal physiological conditions, cell sphere formation may be one of the significant characteristics of robustly proliferative and early-stage stem cells, such as embryonic stem cells forming spheres or colonies on feeder layers or neural stem cells forming neurospheres (Toma et al., 2001). In our self-feeding three-dimensional adipose stem cell (SLF-3D ASC) culture scheme (Figure 1), we take advantage of the inherent proliferative heterogeneity properties of ASCs, allowing a small subset of highly proliferative subpopulations to form cell spheroids on specific matrix scaffolds; this special matrix is formed by a subset of plastic-adherent ASCs. Since the number of cells is very small compared to the whole ASC population, there has been no effective way to screen for these spheroid-forming cells from the ASC population, but our method provides an effective solution. As this method of cell culture does not require other exogenous extracellular matrices or scaffolds, the scheme is simple and economical. In short, SLF-3D spheroid formation in our developed method occurs for the following reasons: 1) highly proliferative cells will grow as spheroids, and 2) scaffolds are formed by the ASCs themselves, providing not only a cell matrix but also nutrition, signaling, and other features, such as growth factors and exosomes.

For the 3D cell culture of mesenchymal stem cells (MSCs), the most common culture method is a scaffold-free 3D culture, which uses ULA culture flasks (Ryu et al., 2019). With this method, all the cells are forced to form cell spheroids because they cannot attach to the plastic dish surface; the spheroids form entirely by adherent cell aggregation after being forced into suspension, unlike neurospheres, which form *via* rapid self-organization after cell proliferation. For this reason, both stem cells and differentiated cells exist among the cell spheroids formed by 3D cell culture. Additionally, when the cell spheroid size increases slightly in the ULA 3D culture, the core of the 3D sphere gradually turns black, and the cells in the center of the spheroid die; this condition leads to a significant reduction in cell numbers and shortened cell passage times. In our newly developed method, the cell spheroids are composed of cells with a strong proliferation capability. In contrast to other 3D culture protocols, our semi-suspended 3D method avoids the use of any artificial physical or chemical method to remove specific subpopulations or senescent cells, resulting in a high yield of ASCs that is maintained for a long time.

In most studies of 3D cell culture methods, researchers have focused on various surface materials, while lacking sufficient attention to the culture medium. In our innovative SLF-3D method, the key to the formation of 3D cell spheres is not the surface material, but the cell culture medium and self-attached ASCs. In classical 3D cell culture methods, the physicochemical composition of the surface material of the culture vessel prevents the cells from being attached to the vessel surface, which directly or indirectly affects the normal physiological state of the cells.

In our innovative SLF-3D culture method, the system contains growth factors and the adherent cell layer, which will provide sufficient specific growth factors and exosomes; consequently, SLF-3D spheroids will be maintained in normal growth and proliferation state. In this case, the cells are very close to their normal physiological state.

For *in vitro* cell culture, the growth factors of the medium have a great influence on the cells. We want to know more about what happens when the ULA-3D spheroid was growing in the SLF-3D medium, but we found that under a low serum medium with EGF, PDGF, and bFGF, over 5 days, the death rate of ULA-3D cells will increase sharply (Supplementary Figure S2). We speculate that ULA-3D spheroids may require more nutrients in its abnormal physiological state, that is, suspended growth state, and that 3% FBS does not provide its required nutrients. In addition, as to why SLF-3D spheroids can survive, we believe that the ASCs are adapted to low-serum starvation for one to two passages before SLF-3D spheroid formation; therefore, the SLF-3D spheroids can survive and proliferate normally under low serum conditions.

It has been reported that a variety of ECM substitutes can have a dramatic impact on cell growth, proliferation, and even life and death (Gospodarowicz et al., 1980; Zhang et al., 2009; Wong et al., 2018; Dolega et al., 2021). We first screen four ECM genes from common ECM-related genes, and then checked the expression level of these genes in SLF-3D and ULA-3D spheroids on 0, 3, and 7 days (Figure 7). We found that the trend of the expression level of MMP1, MMP3, HAS1, and FN1 was different. Among them, the expression level of HAS1 and MMP1 increased and the expression trend was different from ULA-3D at 7 days. In addition, it has been reported that the Hyaluronan (HA) plays an important role in ASC proliferation (Chen et al., 2007; Flynn et al., 2008), and the MMP1 influenced cell proliferation in epithelial cells and neural progenitor cells (Herrera et al., 2013; Valente et al., 2015). All these pieces of evidence strongly hinted that the elevated expression of HAS1 and MMP1 of SLF-3D may be one of the reasons for maintaining the proliferation and less necrosis of SLF-3D spheroids.

In conclusion, the results of this study provide a set of feasible methods for culturing 3D ASC spheroids with relatively uniform properties and certain proliferation capacity, and we believe that this novel 3D culture method has potential for use in a wide variety of clinical applications, such as stem cell culture, tissue engineering, and drug screening.

## Data Availability

The original contributions presented in the study are included in the article/Supplementary Material, further inquiries can be directed to the corresponding authors.

## Abbreviations

αSMA, α-smooth muscle actin; CHSY, chondroitin synthase 1; Col1α, collagen type 1a; Col3α; collagen type 3α; Col5α; collagen type 5α; ELN, elastin; FN1, fibronectin1; HAS, hyaluronic acid/hyaluronan synthase; LAMA1, laminin alpha1; LAMB1, laminin β‐1; MMP1/2/3, Matrix metallopeptidase 1/2/3; ZO1, zona occludens 1.
