# Supplementary material for: A Novel 3D Culture Model of Human ASCs Reduces Cell Death in Spheroid Cores and Maintains Inner Cell Proliferation Compared With a Nonadherent 3D Culture
Source: Front Cell Dev Biol. 2021 Nov 11;9:737275. doi: 10.3389/fcell.2021.737275 (PMC8632442; doi:10.3389/fcell.2021.737275)
Supplement: Supplementary file 1 [file DataSheet1.PDF]

Supplementary Materials

Materials and Methods

The inducted differentiation of human ADSCs and self-feeder 3D ASC (SLF-3D)

For adipogenic, osteogenic, and chondrogenic differentiation, approximately 80–90% confluent ADSCs were grown in 24-well cell culture plates precoated with a 0.1% gelatin solution. Then, ADSCs were incubated with adipogenic differentiation induction medium (Cyagen Bioscience, Inc., Guangzhou, China) for 2 weeks, or osteogenic and chondrogenic differentiation induction medium (Cyagen Bioscience, Inc., Guangzhou, China) for 3 weeks, respectively. ADSCs induced by adipogenic, osteogenic, and chondrogenic differentiation were fixed with 4% paraformaldehyde and stained with Oil Red O, Alizarin Red S, or Alcian blue to detect the results of inducement culture. Images were observed under a Zeiss Observe A1 light microscope with an Axiocam 506 colour digital camera (Carl Zeiss, Germany).

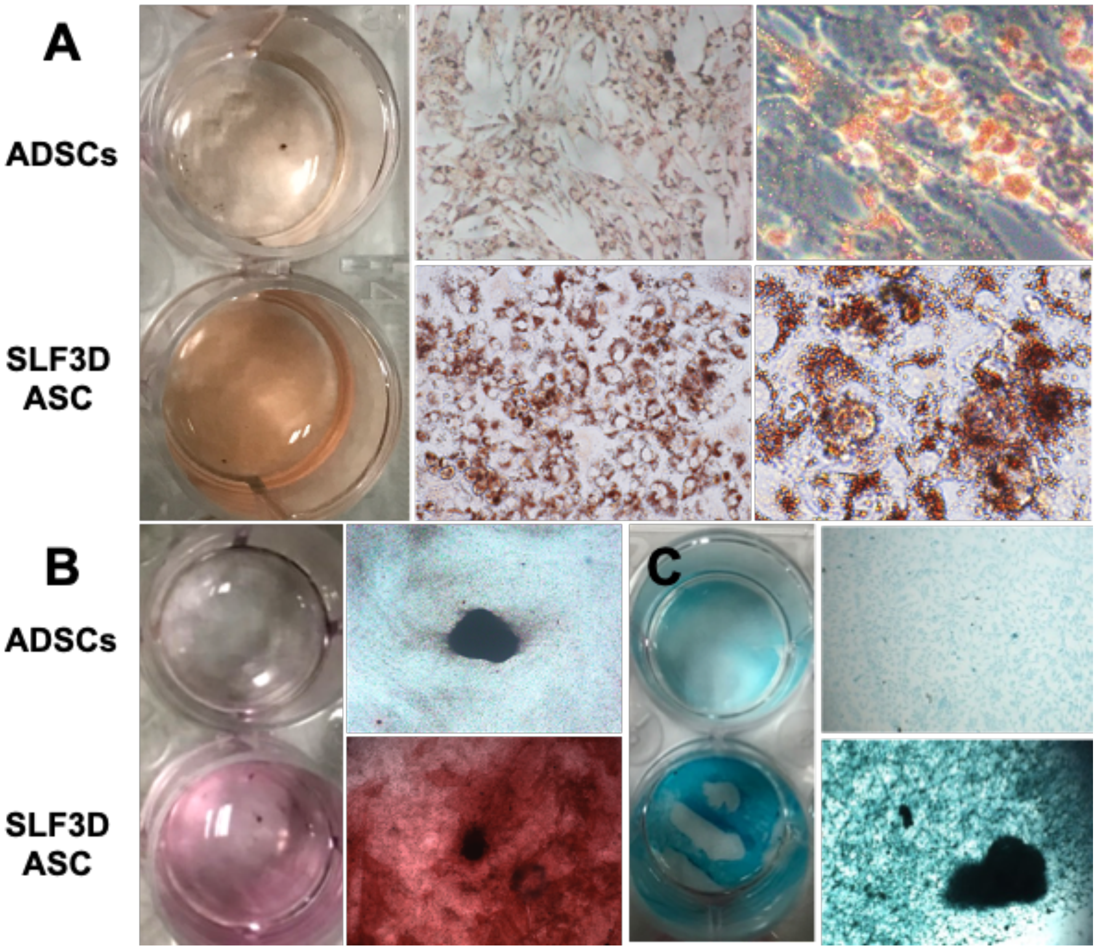

Fig. S1. Characterization of SLF3D ASC and ADSCs. Staining for adipogenic, osteogenic, and chondrogenic differentiation of SLF3D ASC and ADSCs. Oil red O staining of cells cultured for 14 days with adipogenic differentiation medium (A). Alizarin red staining of cells cultured for 21 days with osteogenic differentiation medium (B). Alcian blue staining of cells cultured for 21 days with chondrogenic medium (C).

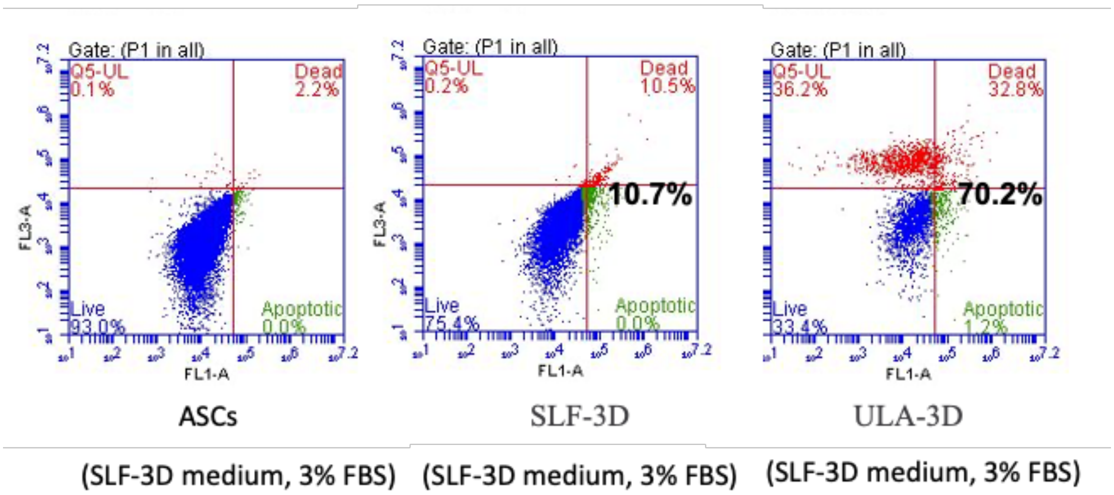

Fig. S2. In SLF-3D medium, the cells viability of SLF-3D and ULA-3D spheroid were evaluated by flow cytometry.

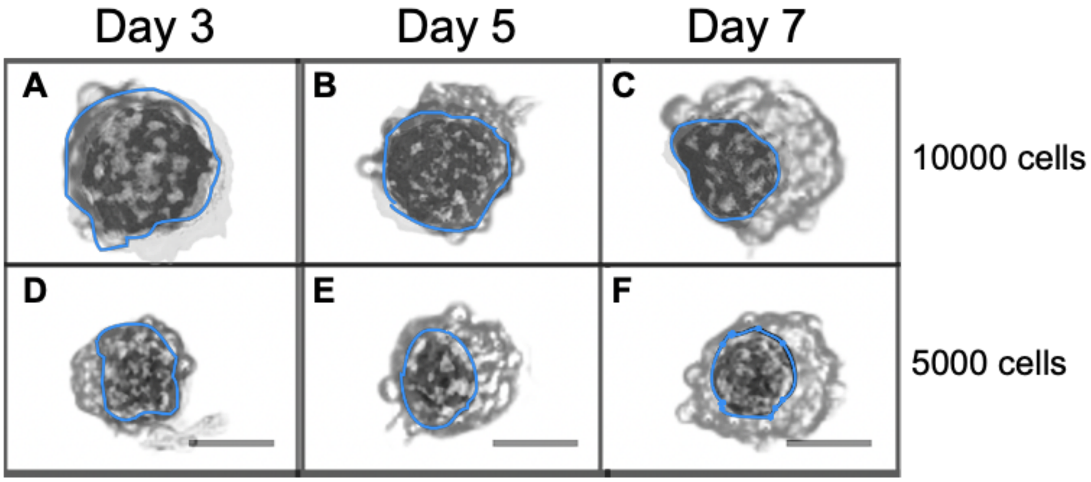

Fig. S3. Comparison of the morphology of SLF-3D and ULA-3D spheroid on different culture time.

The spheroids generated by the self-feeder 3D (SLF-3D) culture method were compared with the spheroids from the traditional ultra-low adhesion 3D (ULA-3D) method on days 3, 5, and 7. For compared SLF-3D and ULA-3D spheroids, 10000 cells (A, B, C) or 5000 cells (D, E, F) formed spheroids were binned and overlapped on the figure, the 3D cell spheroids marked with outline lines are the ULA-3D cell spheroids (scale bars, 100 μm). The details are described in the “Materials and method” section.
